# Characterization of Growth Performance, Pork Quality, and Body Composition in Mangalica Pigs

**DOI:** 10.3390/foods12030554

**Published:** 2023-01-26

**Authors:** Morgan M. Roberts, Stephanie D. Perkins, Brian L. Anderson, Jason T. Sawyer, Terry D. Brandebourg

**Affiliations:** Department of Animal Sciences, Auburn University, Auburn, AL 36849, USA

**Keywords:** growth performance, Mangalica, marbling, pork quality, body composition

## Abstract

European heritage breeds, such as the Blonde (B), Red (R), and Swallow-bellied (SB) Mangalica pig, display an extreme propensity to fatten and are reputed to produce superior quality pork. This suggests that Mangalica pork should command a higher price, and the Mangalica is a candidate breed to target niche markets within the United States. Our objectives were to test this hypothesis by (1) directly comparing growth performance and carcass merit of purebred Yorkshire (Y), B, R, and SB Mangalica pigs to identify the best breed for adoption, and (2) comparing indices of pork quality in purebred R, Y, and crossbred (R × Y) pigs to determine if crossbreeding represented a viable alternative to the adoption of purebred Mangalica. Daily feed intake, average daily gain (ADG), and feed efficiency were highest in Y and lowest in SB pigs with B and R ranked intermediately (*p* < 0.001). Backfat thickness was greatest in B and lowest in Y with R and SB ranked intermediately (*p* < 0.001). Marbling score was greatest in R pigs and lowest in Y pigs with B and SB ranked intermediately (*p* < 0.01). In contrast, loin eye area (LEA) was greatest in Y pigs compared to B, R, and SB (*p* < 0.001). Indices of meat quality were then compared in R, R × Y, and Y pigs. Backfat thickness and marbling scores were greater in R than R × Y and Y pigs (*p* < 0.001) while LEA was greater in Y than R × Y and R pigs (*p* < 0.001). Loin and ham ultimate pH, color, and firmness scores were significantly greater in R than R × Y or Y pigs (*p* < 0.05). Meanwhile, cook loss was significantly less in R than Y pigs (*p* < 0.007) while Warner-Bratzler Shear Force (WBS) was not different in chops between groups (*p* < 0.11). These data indicate that though Mangalica exhibit poorer growth performance, Mangalica pork exhibits superior pork quality attributes, suggesting that higher price points for Mangalica pork in niche markets are justified.

## 1. Introduction

Selection for rapid growth and higher feed efficiency within the United States pork industry has tended to negatively impact important pork quality traits, such as flavor, juiciness, tenderness, color, and water-holding capacity [1,2,3]. This in turn has led to growth in niche markets whereby consumers are willing to pay premium prices for what is perceived as exceptional pork eating experiences. Within U.S. markets, the two most important quality attributes influencing consumer choice at the point of sale are the color and degree of marbling displayed by pork cuts both being associated with enhanced tenderness by consumers [2,4,5,6,7]. Thus, breeds adopted to target U.S. niche markets should produce pork cuts with redder color, superior marbling, and enhanced tenderness while displaying growth performance that does not limit the economic viability of adopting the breed [2,4].

European heritage breeds such as the Mangalica pig appear to display the pork quality characteristics necessary to meet these emerging market opportunities within the U.S. [8,9]. The resurgence of the European Mangalica from near extinction has fostered significant research into this pig both due to its cultural significant and its desirable use as a food animal. The Mangalica encompasses three closely related breeds distinguished by their Blonde (White), Red, and Swallow-bellied (Black with golden underbelly) coat colors with each also displaying apparent differences in their carcass characteristics [8,10]. Originally domesticated to fulfill a need for lard production, the Mangalica is slow growing, displays poor feed efficiency, and produces carcasses that can exhibit as little as 30% lean and as much as 70% fat at typical harvest weights [8,9,10,11,12,13,14,15,16,17,18,19,20,21,22,23,24,25]. In contrast, the genetically improved Yorkshire pig, a breed closely related to the European Large White, produces carcasses that are approximately 70% lean and only 30% fat while displaying high feed efficiency, rapid lean growth, and high reproductive performance [26,27]. Such phenotypic traits have positioned the Yorkshire as the most popular breed of pig for pork production in the United States though it displays significantly less marbling and often produces paler pork cuts than other breeds [26,27].

The Mangalica’s reputation for producing superior pork appears well founded. Multiple studies that have compared either Blonde or Swallow-bellied Mangalica directly with the Large White indicate that both Blondes and Swallow-bellied Mangalica display higher degrees of marbling and their muscles appear redder than their modern counterparts [20,21,22,23,24]. Interestingly, Mangalica pork contains a higher degree of monounsaturated fatty acids than the Large White, further suggesting that the Mangalica is uniquely positioned as a food animal both in terms of the nutritive quality of its fresh pork and its potential to yield dry cured products with distinctive flavor [22,23,24,28]. Furthermore, crossing purebred Mangalica with European Landrace/Yorkshire sows appears to be a viable strategy to produce pork with desirable sensory attributes [29].

Given that Mangalica pigs display the pork quality characteristics necessary for targeting niche markets, the aims of this study were to (1) directly compare differences in growth performance and body composition between the three Mangalica breeds, and (2) determine if crossbreeding represented a viable alternative to the adoption of purebred Mangalica. These aims were accomplished by performing growth trials in a modern intensive production system typical of the U.S. pork industry to directly compare the growth performance and pork quality attributes of Blonde (B), Red (R), and Swallow-bellied (SB) Mangalica to Yorkshire (Y) pigs. Subsequently, indices of pork quality were furthered compared between R, Y, and Red/Yorkshire (R × Y) crosses. The obtained data will allow pork producers targeting niche markets within the United States to make more knowledgeable decisions concerning breed adoption and breeding strategies.

## 2. Materials and Methods

### 2.1. Animals and Design

All experimental procedures were approved by the Auburn University Institutional Animal Care and Use Committee (IACUC; approval number PRN 2011-2001). The Auburn University College of Agriculture is accredited by the Association for Assessment and Accreditation of Laboratory Animal Care International (AALAC) and this study was conducted in accordance with the Federation of Animal Science Societies’ Guide for the Care and Use of Agricultural Animals in Research and Teaching. Purebred Yorkshire pigs, red, blonde, and swallow-bellied lines of Mangalica pigs were housed individually in 12.2 m^2^ pens at the Auburn University Swine Research and Education Center (SREC) for the duration of these experiments. In addition, Yorkshire boars were crossbred to red Mangalica sows to yield York × Red crosses. All pigs were fed a typical grower ration (17% CP) from 18 to 54 kg body weight (BW) and a finisher ration (15% CP) from 55 to 110 kg BW. Pigs were provided ad libitum access to water. Daily feed intakes and weekly body weights were recorded during a period spanning growth from 18 kg live weight until reaching harvest weight of 110 kg to facilitate measurement of average daily gain (ADG), feed efficiency (kg gained/kg feed), and total feed intake. 

### 2.2. Carcass Fabrication

Animals were harvested at the Auburn University Lambert-Powell Meats Lab under USDA-FSIS inspection. Hot carcass weight was recorded after harvest, and carcasses were chilled at 2 ± 1 °C for 24 h. At 24 h postmortem, carcass pH was recorded in the left side ham using a pH Spear probe (Oakton Instruments, Vernon Hills, IL, USA). Both live weight and hot carcass weight were recorded to determine dressing percentage. Following the 24 h chill period, carcasses were ribbed between the 10th and 11th rib. Backfat was measured at the 10th and last rib, and loin eye area (LEA) was also measured by trained personnel. Backfat and LEA were adjusted based on the recommended equation of National Pork Producer’s Council (NPPC) [3]. The following equations were used: Adjusted Backfat to 114 kg.= Actual Backfat + [(114-actual wt.) × actual backfat/(actual wt.-114] and Adjusted LEA to 114 kg = Actual LEA + [(114-actual wt.) × actual LEA/(actual wt. + 70)].

Evaluation of subjective scores for marbling, firmness, and muscle score were determined by a trained observer using published visual standards [3]. Additionally, the longissimus muscle at the 10th rib was evaluated for objective color measurements with a Hunter Miniscan XE Plus (Hunter Lab, Reston, VA, USA) to determine Hunter L*, a*, and b* values. The Miniscan was calibrated according to the manufacturer’s recommendations and utilized a D65 light source, a 10° viewing angle, and a 35-mm viewing area. Following carcass evaluation, a section of the longissimus muscle was removed from the 11th rib to the last lumbar vertebrae from each carcass for meat quality analysis.

### 2.3. Warner-Bratzler Shear Force and Cook Loss

Warner Bratzler shear force (WBS) evaluation was performed using pork loin chops cut to 2.54-cm thickness, thawed at approximately 4 °C for 24 h. Chops were weighed and placed on a Calphalon Removable Plate Grill (Calphalon, Perrysburg, OH, USA) clamshell style contact grill pre-heated to 177 °C. Temperature was monitored with a copper constantan thermocouple wire inserted into the geometric center of the chop and attached to a hand-held Omega data logger HH309A (Omega, Stamford, CT, USA) temperature recorder. Chops were cooked for 7 min to an internal temperature of 71 °C. Cooked chops were removed and reweighed before being placed on non-absorbent wax-coated paper to cool to room temperature. After cooling chops were wrapped in aluminum foil and placed in refrigerator at 4 °C for approximately 24 h. Following refrigeration, six 1.27cmdiameter cores were removed from each chop with a brass cork borer (Model 1601A Series Brass Cork Borer, Boekel Scientific, Feasterville, PA, USA) parallel to the longitudinal orientation of the muscle fibers. Each core was sheared once at its center using a Warner-Bratzler Shear force blade attached to a TA-XT2i Texture Analyzer (Texture Technologies Corp., Scarsdale, NY, USA) with a crosshead speed of 200 mm/min. The peak force measurements were averaged from the six cores of each sample and used for analysis. The probe was programmed to be lowered 30 mm after detection of resistance. The penetration speed was 3.3 mm/s with a post-test speed of 10 mm/s and a pre-test speed of 2.0 mm/s. Peak force of each sample was obtained and expressed in Newtons (N). Cook loss was measured as the percent of pre-cooked weight lost during cooking and calculated as Cook Loss = (Initial weight – Cooked weight) × 100.

### 2.4. Gene Expression Analysis

Upon exsanguination, subcutaneous adipose tissues were immediately collected, snap frozen in liquid nitrogen, and stored at −80 °C until mRNA analysis. Total RNA was extracted from adipose tissue using a two-step purification protocol with total RNA first being extracted from whole tissue using RNAzol^®^ RT (MRC, Inc, Cincinnati, OH, USA) followed by purification using RNAeasy spin columns (QIAGEN, Inc., Valencia, CA, USA) according to the manufacturers’ recommendations. RNA was quantified using a BioTek Synergy 4 plate reader utilizing the Take3 system (BioTek U.S., Winooski, VT, USA) with all samples exhibiting an OD 260/280 between 1.8 and 2.0 and an OD 260/230 value between 1.8 and 2.2. Spectral scans ranging from 200 to 400 nm further verified sample purity as all RNA samples produced smooth curves exhibiting one peak at 260 nm. Total RNA integrity was accessed both visually by resolving 2 µg of RNA on a denaturing formaldehyde gel containing ethidium bromide and by determining an RNA Integrity Number (RIN) using an Agilent 2100 bioanalyzer (Agilent Technologies, Inc., Clara, CA, USA). All samples demonstrated sharp ribosomal bands with a 28S to 18S ratio greater than 1 and RIN values greater than 7.0 and were thus judged intact and non-degraded. Total RNA was then reverse transcribed using Superscript II reverse transcriptase (Promega Inc, Madison, WI, USA) and oligo-dT primers. Real-time PCR was performed on the resultant cDNA using a Roche Lightcycler^®^ 480 Real-time PCR machine and LightCycler^®^ 480 SYBR Green I Master Mix (Roche Applied Science, Indianapolis, IN, USA) according to manufacturer’s recommendations. All PCR reactions were performed using intron-spanning primers under optimized conditions with primer efficiencies ranging between 90 to 101% as verified with standard curves. Product purity was assessed by melting curve analysis and expected amplicon sizes were verified on a 2% agarose gel stained with ethidium bromide. Values were normalized to Ribosomal Protein S15 (S15) mRNA expression. The S15 mRNA levels represent an appropriate control as the efficiency of the S15 primers was 100% and S15 mRNA expression was not different between any groups tested (*p* < 0.93). Data are expressed as fold change relative to baseline and calculated according to Pfaffl, 2010 [30].

### 2.5. Statistical Analysis

Changes in gene expression were calculated from the cycle threshold, after correction using S15 expression and analyzed using the Pair Wise Fixed Reallocation Randomization Test of REST-MCS v2.0 (http://rest.gene-quantification.info/ (accessed on 3 October 2022). Carcass traits and RNA quality data were analyzed as a completely randomized block design using a mixed linear model of SAS v9.2 with individual animal serving as the experimental unit, i.e., individual block (SAS Institute, Inc., Cary, NC, USA).

## 3. Results

**Figure 1:**
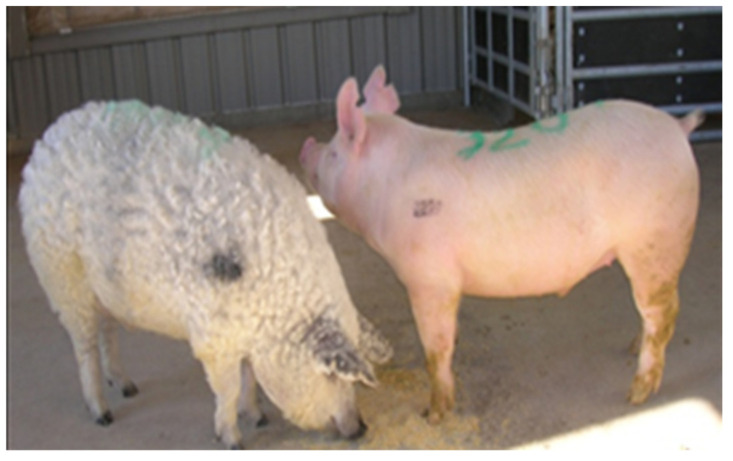
Comparison of weight matched Blonde Mangalica (left) and Yorkshire (right) pigs at 115 kg live weight.

**Figure 2:**
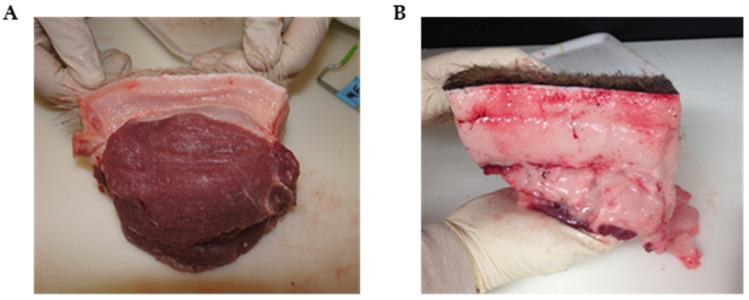
Depiction of differences in subcutaneous fat development and thickness at 115 kg harvest weight between Yorkshire (**A**) and Blonde Mangalica (**B**) pigs. Tissue was harvested immediately following exsanguination by dissecting an 8 cm deep plug of tissue over the tenth rib from the skin to the underlying *longissimus* muscle. Three distinct adipose tissue layers are visible on the Mangalica sample while the much thinner Yorkshire sample is devoid of the inner layer of subcutaneous fat and exhibits much thinner existing layers compared to the Mangalica.

**Figure 3:**
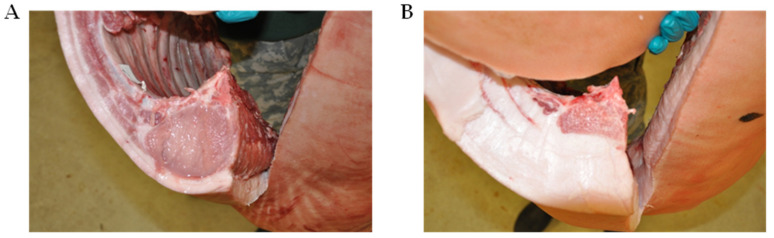
Depiction of differences in back fat and loin eye area on carcasses at 115 kg harvest weight between Yorkshire (**A**) and Blonde Mangalica pigs (**B**). Carcasses were split at the tenth rib to measure backfat thickness and loin eye area following storage at 4 °C for 24 h. These representative images are consistent with carcass data reported in Table 1.

Pigs were randomly assigned to individual pens at 18 kg live weight and allowed ad libitum access to typical growth stage-matched rations until pigs reached approximately 54 kg live weight. Growth performance was monitored throughout this period and body composition compared at harvest for Yorkshire pigs and the three true breeding varieties of Mangalica pigs. As depicted in Figure 1, Mangalica and Yorkshire pigs displayed obvious phenotypic differences with Mangalica representing a lard-type body composition and Yorkshire pigs resembling modern, lean, and muscled pigs. As shown in Table 1, all indices of growth performance and body composition measured were significantly different between Yorkshire and Mangalica pigs. At market weight, Mangalica pigs were significantly fatter, exhibiting both greater backfat at the 10th rib (*p* < 0.0001) and significantly greater amounts of marbling within the longissimus muscle than Yorkshire pigs (*p* < 0.01). However, Mangalica pigs developed significantly less muscle mass at matched weights than Yorkshire pigs as estimated by *Longissimus dorsi* muscle area (LEA) which was greater than twice the size in Yorkshire carcasses compared to the three Mangalica breeds (*p* < 0.0001). Likewise, muscle score values were consistent with LEA measurements (*p* < 0.01). These differences in adiposity and muscling were so extreme as to be visually appreciable post-harvest (Figure 2 and Figure 3). Consistent with differences in carcass composition, Yorkshire pigs grew faster as evidenced by higher average daily gains (*p* < 0.0001) and exhibited superior feed efficiency (*p* < 0.0001) compared to Mangalica pigs, despite Yorkshires displaying slightly higher voluntary feed intake levels (*p* < 0.05). 

Differences in growth and body composition parameters were also observed between the three Mangalica pig breeds (Table 1). Blonde Mangalica were fatter than their Red and Swallow-bellied counterparts based upon subcutaneous fat thickness (*p* < 0.05). Interestingly, Red Mangalica exhibited significantly higher degrees of marbling than other Mangalica types (*p* < 0.05). Swallow-bellied Mangalica exhibited lower average daily gains (*p* < 0.05) likely due to their lower voluntary intake (*p* < 0.05) and lower muscling (*p* < 0.05) compared to their Blonde and Red counterparts.

**Figure 4:**
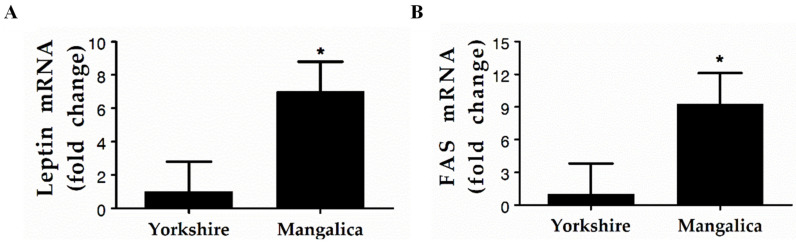
The expression of leptin and fatty acid synthetase (FAS) mRNA as markers for adiposity in lean Yorkshire and obese Mangalica pigs. (**A**): Leptin mRNA levels in subcutaneous adipose tissue. (**B**): FAS mRNA levels in subcutaneous adipose tissue. The mRNA levels were measured using real-time PCR. Expression levels were normalized relative to the porcine S15 gene and are presented as fold change relative to expression in lean pigwith * denoting *p* < 0.05. *n* = 8 per treatment.

To determine if the divergent phenotypes were associated with meaningful physiological differences, leptin, and fatty acid synthetase (FAS) mRNA was measured in subcutaneous adipose tissue of market weight Yorkshire and Blonde Mangalica pigs using real-time PCR (Figure 4). Consistent with the obese phenotype of the Mangalica, leptin mRNA expression was 6.8-fold higher and FAS mRNA expression was 9.2-fold higher in the subcutaneous adipose tissue of Mangalica versus Yorkshire pigs (*p* < 0.01). 

Indices of meat quality were compared in Red Mangalica, Red Mangalica × Yorkshire, and Yorkshire pigs, and the data are presented in Table 2. There were no differences in live weight between groups, indicating that differences in carcass parameters were not confounded by slaughter weight (*p* < 0.98). However, back fat thickness was 1.8-fold and 3.4-fold greater in Red Mangalica than Red Mangalica × Yorkshire and Yorkshire pigs (*p* < 0.0001). Marbling score was 1.5 and 2.8-fold greater in Red Mangalica than Red Mangalica × Yorkshire and Yorkshire pigs (*p* < 0.005), and LEA was 1.5-fold and 2.3-fold greater in Yorkshire than Red Mangalica × Yorkshire and Red Mangalica pigs (*p* < 0.0001). Muscle score displayed the same pattern as LEA (*p* < 0.05). Firmness scores were highest in Red Mangalica and lowest in Yorkshire pigs (*p* < 0.03). Loin and ham ultimate pH was significantly greater in Red Mangalica than Red Mangalica × Yorkshire or Yorkshire pigs (*p* < 0.01) mirroring color (*p* < 0.005) and firmness scores (*p* < 0.003). Cook loss was significantly lesser in Red Mangalica than Yorkshire pigs (*p* < 0.007) while WBS was not different in chops between groups (*p* < 0.11).

## 4. Discussion

The Mangalica pig is an interesting heritage breed that was once prized in Hungary for its lard production and superior meat quality [7]. Unlike the modern Yorkshire, which has undergone intense, methodical genetic selection for rapid, lean growth within the U.S., the Mangalica genetics reflect a general lack of selection pressure [15,16,17]. Therefore, Mangalica genetics are best described as remaining primitive or unimproved relative to the breed’s derivation which has served to preserve distinctive carcass quality traits that distinguish their phenotypes from improved meat breeds [31]. There are three breeds of Mangalica pigs that survive today, the Red, Blonde, and Swallow-belly [8,10,15,17]. The Mangalica breed was first established in the 1830s when efforts were undertaken to create a unique lard-type hog which displayed high quality fat [8,15,17]. Initially, the Sumadia pig was crossbred with Hungarian aboriginal breeds, the Alföldi and the Bakony, to create the Blonde and Black Mangalica. The Blonde and Black were then crossbred to form the Swallow-Belly Mangalica. The Blonde was also crossbred with another aboriginal breed, the Szalonta, to generate the Red Mangalica. The Blonde coat color can range from shades of light gray to yellow, while the Swallow-belly is black with a yellow/blonde throat and underbelly. The Red Mangalica is ginger in color with a slightly thinner and slicker hair coat [8]. All three pigs have been prized for their hardiness and high-quality lard though apparent differences in their carcass characteristics persist [8,9,10,11,12,13,14,15,16,17,18,19,20,21,22,23,24,25].

In the current study, Yorkshire pigs exhibited an ADG of 0.97 kg/day, a voluntary feed intake (FI) of 2.71 kg/day and a feed efficiency of 0.357 for the period on test. Expected industry performance for purebred Yorkshire herds in the U.S. over similar growing periods as this trial range between 0.77 and 0.88 kg/day for ADG, 1.95 and 2.5 kg for daily feed intake, and 0.31 and 0.45 for feed efficiency [26,32]. Yorkshire pigs in the current study exhibited tenth rib back fat thickness of 2.34 cm and a LEA of 16.56 cm^2^ at market weight compared to an industry standard of 2.29 cm backfat and LEA of 15.7 cm^2^. Thus, Yorkshire pigs in the current study gained faster but also consumed more feed than typical herds while exhibiting similar adiposity and slightly greater muscularity compared to the current U.S. industry standard Yorkshire hog. Such differences could be due to differences in breeding stock, management practices, and differences in breeding programs. Moreover, the swine herd at Auburn University is a naïve, closed herd in terms of health status and is reared within a biosecure facility. The lack of immune stimulation in the current rearing facility compared to dirtier environments typical of industry facilities may in part explain the greater growth and feed intake in these pigs, though overall feed efficiency in this study was typical for Yorkshires [33]. Finally, pigs were individually penned so while they were able to socialize with neighbors, they did not have to contend with a dominance hierarchy concerning feeding. Nonetheless, growth performance exhibited by Yorkshire pigs in the current study was consistent with expected ranges for this breed, indicating the Yorkshire pigs in the current study represent a valid baseline in which to compare the three Mangalica breeds.

Growth performance in the Mangalica pig would be expected to be poorer than that of improved breed based upon the literature addressing this issue. Furthermore, the existing studies often characterize Mangalica herds that were reared in what would be considered traditional, pasture-based systems compared to modern, intensive environments. Nonetheless, a survey of such studies indicates that Mangalica pigs exhibit an ADG of 0.25 kg/day, a daily feed intake of roughly 2.3 kg and a feed efficiency of 0.11 [8,9,10,11,12,13,14,15,16,17,18,19,20,21,22,23,24,25]. In the current study, all Mangalica breeds studied significantly exceeded those performance standards. This was expected as Mangalica on this trial were given ad libitum access to concentrated, balanced rations formulated to match their stage of growth with the express goal of maximizing their growth rate and potential to fatten. This is in sharp contrast with the nutrition of Mangalica in traditional growing systems in which a significant portion of their diet is met through foraging on pasture and in woodlots. Two studies examined Blonde Mangalica in intensive production systems and reported ADG ranging from 0.3 to 0.45 kg across the grow-finish phases, which approached growth rates observed in the current study [22,23,24]. The disease-free status of the closed herd in the current study may explain why Mangalica in the current study performed better comparatively. Likewise, differences in genetic backgrounds of the herds utilized may contribute to differences observed between studies conducted in Europe and those utilizing pigs imported in the U.S. Nonetheless, while growth performance was higher for Mangalica in the current study compared to the literature, as expected when compared to Yorkshire pigs, all three Mangalica breeds displayed much poorer growth performance. 

Due to their extreme propensity to fatten, the Mangalica represent an attractive model to study fat development [26,27]. Furthermore, since the three breeds are derived from different lineages [8,9,10,15,17], we hypothesized that breed differences may exist that could be meaningful for producers contemplating adoption of the Mangalica. As expected, Yorkshire and Mangalica pigs demonstrated divergent phenotypes based upon the growth and carcass parameters measured. Importantly, when comparing Mangalica directly, significant differences in growth performance and body composition were indeed observed between the three Mangalica breeds. For instance, in the current study, Red Mangalica displayed the highest ADG and FE of the three breeds. Blonde Mangalica exhibited the highest degree of adiposity based upon subcutaneous fat thickness at the tenth rib. Surprisingly, though, Red Mangalica exhibited the most intramuscular fat of any breed examined. Swallow-bellied Mangalica displayed the lowest ADG and FE while offering no advantages concerning muscle size or marbling compared to the other Mangalica breeds. Given Red Mangalica had superior growth performance and marbling while having similar LEA and less fat cover overall, these data suggest the Red Mangalica offer economic and carcass merit advantages for producers targeting niche breeds compared to either Blonde or Swallow-bellied Mangalica. Upon surveying the literature, these data appear to be the first to directly characterize growth performance, body composition, and meat quality traits in the three Mangalica pig breeds simultaneously. Thus, this study represents a necessary and important contribution to the literature.

While marbling and pork color are critical quality attributes driving the purchasing behavior of consumers [2,3,4,5,6,7], growth performance is also an important consideration for producers given its economic impact and the need for producers to be able to sustainably supply product to niche markets to remain viable [4]. Given this, meat quality traits were directly compared in Yorkshire, Red Mangalica, and Red × Yorkshire crosses to test the hypothesis that crossbreeding represents a viable alternative to adopting purebred Mangalica. As expected, overall muscle growth and fattening were intermediary in the crossbred animals compared to either the purebred Yorkshire or Red Mangalica. When examining pork quality attributes, the F1 crosses displayed higher marbling scores and significantly lower cook loss versus the purebred Yorkshires suggesting an advantage for crossbred pigs. However, color scores in the crossbred animals resembled those of Yorkshire pigs, indicating that crossbreeding diminishes the color advantage associated with pork from purebred Red Mangalica. These results generally agree with studies that have examined quality attributes in Blonde × Duroc crossbred under intensive growing conditions whereby the crossbred animals displayed quality attribute values that were intermediary of their purebred Blonde and Duroc counterparts [22,23,24]. Nonetheless, these data appear to be the first to examine pork quality in Red Mangalica × Yorkshire crossbreds reared under intensive conditions. Data from the current study indicate that marbling can be improved through crossbreeding the Yorkshire with Red Mangalica, but this will be associated decreased lean and no color improvement.

Pork quality continues to be an important issue in the U.S. pork industry. The emphasis on selecting pigs for leanness has resulted in a reduction in pork quality due to a loss of color and intramuscular fat [3]. Color is the most important appearance quality trait affecting the visible appeal of pork to consumers [1,2,3,4,34,35,36]. Marbling is an important sensory trait that contributes to the juiciness and flavor of the product and is another key criterion impacting consumer choice at the meat counter [5,6,7]. Unfortunately, selection for leaner pigs has generally reduced the marbling content, contributing to a less satisfying eating experience by the consumer [1,2,3]. This has led to the creation of niche markets whereby consumers are willing to pay a premium for high quality pork products, especially at high end restaurants [4]. In the current study, Red Mangalica pork exhibited significantly higher marbling, firmness, and color scores, while exhibiting lower cook loss consistent with the perception of juicier chops. Crossbreeding was able to capture some of these quality advantages but not others. Collectively, these data indicate that while Mangalica pigs exhibit poorer growth performance, Mangalica pork displays superior meat quality attributes suggesting higher price points for Mangalica pork in niche markets are justified.

The extreme differences in adiposity that were observed between the Mangalica breeds and the Yorkshire in this study were anticipated. The divergent phenotypes are the apparent result of differences in breed development as historically Mangalica breeding strategies have largely been aimed at preservation or favoring more lard cover and quality while the modern Yorkshire phenotype is the result of the decades of intensive genetic selection for rapid, lean growth [8,26]. These differences were also exhibited in the present study by a greater expression of physiological markers of adipose tissue development, such as leptin and fatty acid synthase mRNA expression in the Mangalica compared to the Yorkshire. Such observations are consistent with studies that have examined differential gene expression within adipose tissue of Mangalica compared to lean swine breeds or that have identified single polymorphisms unique to the Mangalica breed [16,37,38,39,40]. While speculation concerning the precise physiological mechanisms driving the divergent adiposity observed between Mangalica and the Yorkshire pig is beyond the scope of data reported herein, it seems clear that distinct genomic differences exist between these breeds which likely result in significant differences in the biochemical and molecular pathways regulating energy balance, nutrient partitioning, and developmental processes such as adipose tissue accretion. Further studies are needed to characterize the molecular mechanisms underlying these phenotypes. Such new knowledge would yield potentially useful selectable markers that could enable improvements in meat quality and allow better control of adipose tissue growth in the pig. 

## 5. Conclusions

Producers adopting heritage breeds as a strategy to target niche markets need an understanding of the growth properties and carcass merit of the breeds they select to effectively manage and market their hogs. The growth trials herein were conducted to directly compare the phenotypes of Blonde, Red, and Swallow-bellied Mangalica. These data show that Blonde Mangalica exhibited the greatest adiposity, suggesting they represent the most promising breed to be developed as a model to study adipose tissue development in the pig. While all Mangalica breeds demonstrated superior pork quality attributes compared to the modern Yorkshire hog, the Red Mangalica displayed the highest degree of marbling compared to all other pigs in this trial and superior growth performance to either Blonde or Swallow-bellied Mangalica. Crossbred pigs displayed increased marbling but did not exhibit a color advantage while producing less muscled carcasses. The Red Mangalica pig may be more attractive for adoption by producers targeting U.S. niche markets. Finally, these data support the hypothesis that higher price points for Mangalica pork in niche markets are justified.

## Figures and Tables

**Table 1:** Growth performance and body composition of Yorkshire and three Mangalica breeds fed ad libitum ^1,2^.

Variable	Yorkshire	Blonde	Red	Swallow-Belly	SEM	*p*-Value
Live weight, kg	102.1	101.7	101.4	101.5	2.1	0.94
Back fat, cm	2.34 ^c^	6.68 ^a^	5.78 ^b^	5.87 ^b^	0.23	0.0001
Marbling score ^3^	1.20 ^c^	2.53 ^b^	4.17 ^a^	2.67 ^b^	0.55	0.01
Muscle score ^4^	2.23 ^a^	1.50 ^b^	1.53 ^b^	1.25 ^b^	0.29	0.01
LEA, cm^2^	16.56 ^a^	7.87 ^b^	8.06 ^b^	6.91 ^b^	0.48	0.0001
Average daily gain, kg	0.97 ^a^	0.56 ^b^	0.62 ^b^	0.40 ^c^	0.03	0.0001
Daily feed intake, kg	2.71 ^a^	2.31 ^b^	2.43 ^b^	1.91 ^c^	0.10	0.0002
Feed efficiency	0.357 ^a^	0.242 ^b^	0.255 ^b^	0.209 ^c^	0.006	0.0001

^1^ Yorkshire breed has been selected for rapid growth; Mangalica breeds: Blonde, Red, Swallow-belly. ^2^ Values are lsmeans, *n* = 16 per treatment, differing superscripts within a variable denote differences between breeds, *p* < 0.05. ^3^ Subjective Marbling Score: 1 to 2.4 = Devoid; 2.5 to 4 = Traces; 4 to 5 = Slight; etc. ^4^ Muscle score: measured in ½ point increments with 1 = Thin and 3 = Thickest.

**Table 2:** Meat quality traits by group ^1^.

Variable	York	York x Red	Red	SEM	*p*-Value
Live weight, kg	110.6	110.4	111.5	3.3	0.98
Back fat, cm.	1.96 ^c^	3.66 ^b^	6.71 ^a^	0.28	0.0001
Marbling score ^2^	1.62 ^c^	2.93 ^b^	4.45 ^a^	0.39	0.0005
LEA, cm^2^	21.6 ^a^	14.7 ^b^	9.70 ^c^	1.19	0.0001
Muscle score ^3^	2.39 ^a^	1.83 ^b^	1.21 ^c^	0.17	0.05
Firmness ^4^	2.70 ^b^	2.85 ^b^	3.32 ^a^	0.20	0.03
Loin Ultimate pH ^5^	5.36 ^b^	5.54 ^b^	5.86 ^a^	0.11	0.01
Ham Ultimate pH ^5^	5.43 ^c^	5.67 ^b^	5.97 ^a^	0.09	0.003
Color ^6^	3.89 ^b^	3.53 ^b^	4.90 ^a^	0.25	0.05
L*, lightness	60.4 ^a^	60.8 ^a^	51.9 ^b^	1.63	0.002
a*, redness	10.2 ^b^	9.6 ^b^	11.9 ^a^	0.30	0.0004
b*, yellowness	17.4 ^a^	17.3 ^a^	16.0 ^b^	0.50	0.10
Cook loss, %	15.3 ^a^	12.8 ^b^	10.2 ^c^	0.98	0.0071
WBS, N ^7^	3.77	3.43	3.85	0.16	0.18

^1^ Values are lsmeans, *n* = 16 per treatment, differing superscripts within a variable denote differences between groups, *p* < 0.05; ^2^ Subjective Marbling Score: 1 to 2.4 = Devoid; 2.5 to 4 = Traces; 4 to 5 = Slight; etc.; ^3^ Muscle score: measured in ½ point increments with 1 = Thin and 3 = Thickest; ^4^ Firmness: measured in ½ point increments with 1 = Very Soft and 5 = Very Firm; ^5^ Ulimate pH: measured 24 h post-harvest on chilled carcasses; ^6^ Visual (subjective) color score: five-point scale where 1 = very light and pale; 5 = dark red etc. ^7^ WBS: Warner Bratzler Shear Force.

## Data Availability

Not applicable.
